# Prof. J. P. White’s Address

**Published:** 1867-03

**Authors:** 


PROF. J. P. WHITE'S ADDRESS.—The Address to the graduating class in the Buffalo Medical College by Prof. James P. White, will appear in our next number. We regret that it cannot be published in the present issue, since many of our readers, and especially the young men to whom it was directed, are expecting the pleasure of its perusal.

